# Is Set for Variability Performance Dependent on Prior Successful Decoding Attempts for Certain Items? An Item-Level Examination

**DOI:** 10.1080/10888438.2026.2704734

**Published:** 2026-08-18

**Authors:** Valeria M. Rigobon, Laura M. Steacy, Signy Wegener, Noam Siegelman, Donald L. Compton

**Affiliations:** a Australian Centre for the Advancement of Literacy, North Sydney, NSW, Australia; b Australian Catholic University, North Sydney, NSW, Australia; c Department of Educational Psychology, University of Connecticut, Storrs, CT, USA; d Department of Psychology and Department of Cognitive and Brain Sciences, Hebrew University of Jerusalem, Jerusalem, Israel; e Florida Center for Reading Research, Tallahassee, FL, USA; f Department of Psychology, Florida State University, Tallahassee, FL, USA

## Abstract

**Purpose:**

The relationship between set for variability (SfV) performance and various isolated word reading measures in developing readers is high (.60–.80 across studies). SfV skill serves as a second step in the decoding process allowing the decoded form of the letter string to be matched with the stored phonological form. Alternatively, during item-based orthographic learning children produce and perhaps store a decoded form of the word that can be accessed during the SfV task, facilitating performance. In the current study we explore the possibility that this second hypothesis can help explain SfV performance for certain items.

**Method:**

In a sample of 499 children (Grades 2–5, *M* = 9.16 years, 51.3% female, 41.08% White, 40.08% African American, 18.84% from other racial/ethnic backgrounds), item-level analyses were used to explore the possibility that performance on certain SfV items might be dependent on prior successful decoding attempts (SDA) while controlling for important child attributes (e.g. decoding fluency) and word characteristics (e.g. spelling-to-pronunciation transparency).

**Results:**

Some (but not all) words on the SfV task may be dependent on prior SDA. Words that are dependent on prior SDA have significantly fewer phonemes and syllables, and trend toward having fewer letters, lower spelling-to-pronunciation transparency, and larger phonological distances between the mispronunciation and target word.

**Conclusion:**

To correctly complete the SfV task, certain items may require the word to have been previously decoded successfully. These findings have implications for how the SfV-word reading relationship is interpreted and tested moving forward.

For most children, reading an unfamiliar word requires deliberate effort involving the coordination of multiple skills, with the fluency of this process varying greatly depending on the child’s knowledge and reading experience and the complexity of the word (e.g., Siegelman et al., 2020; Steacy et al., 2022). In the earliest stages of word reading development, children lack the orthographic knowledge required to identify words immediately from sight and are thus relegated to the slow and effortful process of translating written words to sound (see Castles et al., 2018; Ehri, 2020). This process, known as the *sine qua non* of reading acquisition (Share, 1995), relies heavily on children engaging in phonological recoding – the sequential translation of individual graphemes into their corresponding sounds – followed by connecting the resulting string of sounds to an existing spoken word (i.e., stored phonological representation) in their vocabulary (see Elbro & de Jong, 2017). While many consider the process of translating the word’s decoded form (also known as its spelling pronunciation) to the phonological form stored in the lexicon to be straightforward, there are numerous instances, especially in opaque writing systems like English, where a faithfully decoded word form does not match its standard spoken form. In many cases, but not all, it is close enough to allow the reader to arrive at the correct pronunciation (see Elbro et al., 2012). This “cleanup” process during phonological recoding is certainly necessary for “irregular” words (e.g., was) in English, but depending on the skill and knowledge of the child this may also be true for “regular” words (e.g., milk), particularly if children make a “schwa insertion” after consonants (e.g., “muh*”* for the m in milk). More generally, Elbro et al. (2012) have argued that phonotactic rules (i.e., “blending” rules) also determine the particular pronunciation of the phonemes in orthographically regular words, suggesting that “recognition from deviant pronunciations may be important to reading of orthographically regular words and not only to reading of irregular words” (p. 343).

To accommodate and clean up this mismatch between the decoded form and lexical pronunciation in English spelling, Gibson and Levin (1975) suggested that developing readers need to adopt a “set for variability.” Venezky (1999) later revived this idea in advocating for the use of phonics instruction for developing readers in English with the important caveat that in order for children to be successful with applying a phonics approach to English they would need a “set for variability” (citing the work of Gibson and Levin): “If what is first produced does not sound like something already known from listening, a child has to change one or more of the sound associations (most probably a vowel) and try again” (p. 232). Elbro and de Jong (2017) and Elbro et al. (2012) offered that this skill, now commonly referred to as set for variability (SfV) or mispronunciation correction, serves as a bridge between decoding and lexical pronunciations and may be an important second step in the decoding process. To better understand the relationship between a child’s ability to clean up the mismatch between the decoded and phonological forms of a word and a child’s reading development, SfV skill has been operationalized in the literature as an oral language task (see Tunmer & Chapman, 1998, 2012) requiring an individual to disambiguate the mismatch between the decoded form of an irregular word and its actual lexical pronunciation. For example, in the task the word wasp is pronounced to rhyme with clasp (i.e. /wæsp/), and the individual must recognize the actual pronunciation of the word to be /wɒsp/. Several studies have reported significant variability across items and children in SfV performance with strong correlations between task performance and word reading skill in developing readers (see Edwards et al., 2025; Kearns et al., 2016; Steacy et al., 2023; Tunmer & Chapman, 2012), as well as between task performance and spelling skill in adults (see Rigobon et al., 2024). In addition, some studies suggest that SfV skill has a causal relationship with early word reading development (Savage et al., 2018, 2024), while others have urged readers to exercise caution when evaluating the evidence supporting this claim (Dawson et al., 2023; Steacy et al., 2023). For instance, it has been suggested that general SfV performance captures more distributed knowledge of the connections between orthography, phonology, and semantics in the reading network (Edwards et al., 2022; Steacy et al., 2023), the same three components that influence the “cleanup” process during phonological recoding, according to the Triangle Model (Harm & Seidenberg, 2004; Plaut et al., 1996). In particular, Edwards et al., (2022) findings support the notion that stronger decoders tend to use their knowledge of various phonology-to-orthography connections in order to respond to the SfV task, while poorer decoders tend to rely strictly on phonology, highlighting the “primacy of decoding skill” in allowing for orthographic facilitation during the SfV task (p. 1253).

The ease with which children correct a mispronunciation when reading, assuming the word is part of their oral vocabulary, is related to the “phonological distance” between their mispronunciation (resulting from phonological recoding) and the correct pronunciation of the word (see Edwards et al., 2022). At the child level, this phonological distance – between the output of phonological recoding and the stored lexical pronunciation – likely varies as a function of the child’s knowledge (e.g., vocabulary, phonological forms, rules of orthographic-to-phonological relationships) and reading experiences, whereas at the word level, attributes such as the consistency of spelling-sound relationships affect phonological distance. Thus, a child’s ability to accurately phonologically recode an unfamiliar letter string, combined with word-specific characteristics, influences the degree of mismatch (i.e., phonological distance) between the decoded form and the phonological representation. In some cases, this mismatch can be substantial, making it challenging, if not impossible, for the child to recognize the decoded output (e.g., the decoded form of tomb would be /tɑmb/, sounding more like bomb with the final consonant enunciated) without activating prior experience with the word’s orthographic form.

In the current study, we are interested in comparing words with larger versus smaller distances between the spelling pronunciation and the stored pronunciation, and how children successfully recognize the latter from the former while performing the SfV task. As part of this investigation, we considered Elbro and de Jong’s (2017) assertion that “the *formation* of spelling pronunciations is an intermediate developmental step between letter-sound decoding and sight word reading which is an accurate way of looking at what is usually called orthographic whole word learning” (p. 170). This suggests that for some SfV items, those with relatively large distances between the spelling and lexical pronunciations (i.e., low spelling-to-pronunciation transparency), the most plausible way to correctly complete the SfV task is to have previously phonologically recoded the word, which may leave a trace of the decoding process to become available for recognition during the SfV task. This idea is consistent with the “redundancy principle” of the lexical quality hypothesis (Perfetti, 1992, 2017; Perfetti & Hart, 2002); namely that high quality word representations stored in the orthographic lexicon contain redundant phonological forms, “one from spoken language and one recoverable from orthographic-to-phonological mappings” of the word (p. 190, Perfetti & Hart, 2002). The redundancy principle suggests that performance on some SfV items may be influenced by whether a child has previously decoded the word and stored a decoded form as part of the representation. Dependence on this prior decoding experience may help explain why some items on the SfV task tend to be more difficult than others, building on relevant child characteristics, word features, and an interaction of the two identified in previous studies.

It is clear from Edwards et al., (2022) findings that longer, more frequent, more transparent, and more concrete words are more likely to be recognized and corrected in the SfV task. However, of the most difficult items from that study’s stimuli, (i.e., correctly answered by <15% of the sample), none would be considered relatively low in all four of those word features, suggesting that item difficulty is likely impacted by a more complex interaction of word features. Furthermore, a three-way child-byword interaction from the same study confirmed that regardless of children’s general word reading ability, poorer decoders’ SfV performance was not significantly impacted by the likelihood of the target word’s spelling being derived from the mispronunciation heard in the task, whereas children with higher performance in both decoding and word reading were more likely to recognize and correct the items in the SfV task if they had higher consistency from mispronunciation to target word spelling. This suggests that stronger decoders are more sensitive to possible variations in sublexical grapheme-phoneme relationships and more likely to rely on orthography during the SfV task to arrive at the correct response, a strategy which may be only be effective for items containing P-O correspondences that a child has encountered before. Together with the low average accuracy rates reported for SfV performance, Edwards et al.’s findings suggest that the role of prior decoding experience in SfV performance may not be easily reduced to effects of a child’s general decoding ability or of a single feature typically associated with reading difficulty, such as frequency or length.

In the current study, we reanalyze Steacy et al.'s (2019) dataset (expanded from an original sample of 103 to 499 subjects) to explore the influence of prior word reading experience on item-level SfV performance, rather than predicting word reading accuracy using SfV and other variables. We modeled the effects of relevant child characteristics (general phonological awareness, decoding fluency, and word reading skill), word features (number of phonemes, spelling-to-pronunciation transparency, and dependence on prior decoding attempts), and item-specific word reading accuracy on SfV performance in a larger sample of children. We consider the untimed SfV task a measure that captures an individual’s ability to access the stored (i.e., lexical) representation of a spoken word from its redundant orthographic-to-phonological mapping (i.e., decoded form) for a given word.

To capture the influence of prior decoding experience on SfV performance, we started by dichotomizing the 40 SfV items into those in which the phonological distance between the spelling and lexical pronunciation was near or far (i.e., its spelling-to-pronunciation transparency). Beyond our initial assumption that words with relatively low spelling-to-pronunciation transparency would be more difficult to recognize and correct in the SfV task, we used theoretically driven holistic scoring to determine if a word was dependent on a prior successful decoding attempt (SDA) to recognize and correct in the SfV task. The scoring procedure accounted for three additional relevant word features that may be related to a task item’s dependence on prior SDA: (1) relatively low phonological similarity between the stimulus (decoded pronunciation) and the target response (lexical pronunciation); (2) relatively high maximum phonological-orthographic (P-O) surprisal,^1^ which represents less likely pairings between phonemes in the stimulus and their corresponding graphemes in the target word’s spelling; and (3) relatively large phonological distance between the target word and its nearest phonological neighbors. Items with higher surprisal (i.e., less likely pairings between a phoneme in the stimulus and its corresponding grapheme in the target word’s spelling) could be more difficult to actively convert or “translate” from phonemes to graphemes (i.e., leading participants to rely more heavily on orthographic knowledge from a stored representation to arrive at the correct phonological form). In contrast, when presented with items with lower surprisal in the SfV task, participants could use knowledge of typical phonics and decoding rules to actively convert phonemes heard in the stimulus to the graphemes in the corresponding target word. Similarly, items with more distant phonological neighbors (i.e., more transformations needed to make the next closest phonological neighbors) may be more dependent on prior SDA to recognize them from the mispronunciations in the task as beginning readers cannot rely on the correctly pronounced segments in the task stimulus to activate phonological representations of several highly similar-sounding real words from oral vocabulary. Items closer in pronunciation to their phonological neighbors, however, may activate more real words from oral vocabulary that contain one or more of the correctly pronounced segments from the task stimulus, suggesting that prior experience with decoding these words might not be necessary to recognize and correct them in the SFV task.

Having child-level item performance on both word reading accuracy and SfV performance allowed us to test the probability of successfully completing the SfV task conditionalized on item reading accuracy and whether the item was considered to require prior SDA. Given the holistic approach to creating the prior SDA dependence variable, we examined the properties of words coded as dependent on prior SDA and conducted alternate models to consider other possible explanations for the effects of our SDA coding on SfV performance. Together, findings from the various models offer implications for our understanding of what exactly has been measured by SfV in previous studies and which word features may drive the difficulty of certain SfV items.

## Method

### Participants

Participants were 499 children in Grades 2–5 from private and public schools in the U.S. across several waves of data collection (see Edwards et al., 2022; Steacy et al., 2023). Prior to the study, ethical approval was obtained from the institution’s ethics committee in compliance with the U.S. Federal Policy for the Protection of Human Subjects. Prior to participation, teacher and parent consent was sought and student assent was obtained. Demographic data for the sample are presented in Table 1. Struggling readers were intentionally oversampled from specialized schools for children with learning differences (e.g., dyslexia) or schools that were receiving additional federal funding to support a large population of students from low socioeconomic backgrounds (i.e., Title I schools). However, even with this sampling plan, the overall sample scored within the average range on standardized measures of word identification (*X* = 101.53, *SD* = 13.16), word attack (*X* = 100.85, *SD* = 10.30), sight word reading efficiency (*X* = 93.93, *SD* = 15.91), and phonemic decoding efficiency (*X* = 90.41, *SD* = 15.51) from the Woodcock–Johnson III Tests of Achievement (Woodcock et al., 2001) and Test of Word Reading Efficiency (TOWRE-2; Torgesen et al., 2012), respectively.

### Procedures

Trained research assistants administered all tests in the fall or spring of Grades 2–5. All research assistants received extensive training and practice and were required to achieve ≥80% procedural fidelity before testing participants. All testing sessions were audio recorded for scoring and reliability purposes, followed by double scoring and double data entry for all tests administered in the study. Any remaining discrepancies were resolved by a third trained research assistant.

### Measures

#### Set for Variability (SfV) - (dependent measure).

Based on the work of Tunmer and Chapman (1998, 2012) and Steacy et al. (2019) with elementary-aged students, SfV was evaluated by participants’ ability to derive the correct pronunciation from 40 spoken English words that are “mispronounced” based on regular decoding rules, as they might be if they were regular words or partially decoded^2^ (see Appendix A). The regularized pronunciations had phoneme substitutions or phoneme insertions (e.g., the practice item breakfast mispronounced as /brikfəst/). The task instructions were: “Today we’re going to play a word game with Alex. In this game, Alex tries to say a word but he says it the wrong way. You have to figure out what Alex is trying to say. When Alex says a word, you try to guess what he is trying to say.” Task instructions and mispronunciations were presented as recordings to ensure that all participants heard the same stimuli from a uniform voice in the task, and participants were only allowed to hear the recording one more time if they reported not being able to hear it the first time. If a participant asked to hear the recording again more than once or was not sure of their answer, the tester encouraged the participant to try their best with providing a response before moving on to the next item. Coefficient alpha for this sample was .93. Each response to each word in the SfV task was scored as 0 for incorrect or 1 for correct (i.e., correct target word said out loud).

### Child measures

#### Decoding fluency.

Participants were asked to read a list of 66 nonwords in English as quickly and accurately as possible within the span of 45 s in the Phonemic Decoding Efficiency subtest from the TOWRE-2 (Torgesen et al., 2012). Performance ranged from 0 to 56 in this sample. The authors report an alternate forms reliability of .92.

#### Expressive vocabulary.

The vocabulary (untimed) subtest from the WASI (Wechsler, 2011) required students to identify pictures and define words. Performance ranged from 1 to 39 in this sample. The interrater reliability range for elementary age children is .92–.94 (McCrimmon & Smith, 2013).

#### Phonological awareness (PA).

In the untimed Elision task from the Comprehensive Test of Phonological Processing (CTOPP; Wagner et al., 2013), participants were asked to delete phonological units from words, with performance ranging from 0 to 34 in this sample. The authors report test-retest reliability of .93.

#### Word reading.

In the untimed word identification subtest from the Woodcock–Johnson III (Schrank, 2005), participants were asked to read 76 increasingly difficult words, with performance ranging from 23 to 67 in this sample. The authors report split-half reliability of .94.

#### Word reading (item-specific).

Participants were asked to read the same 40 words from the SfV task. This was an untimed, experimenter created task and Cronbach’s alpha for this sample was .98. Accuracy for each item (0 or 1) represents the item-specific reading measure, in contrast to the total scores on the previously described measure, which represents general word reading ability.

### Word measures^3^

#### Prior Successful Decoding Attempt (SDA) dependence:

Each word in the dependent SfV measure was scored dichotomously (0/1) based on holistic judgments of whether the word would need to be decoded successfully prior to the testing session in order for the student to recognize the word from its mispronunciation and provide its correct pronunciation in the SfV task (see Appendix A). Scorers with knowledge of English phonics and decoding rules agreed to follow four key theoretical assumptions of word features that influence the distance between a decoded pronunciation and the lexical pronunciation, but a word did not need to meet a specific combination of cutoff values in all four of these features to be coded as 0 versus 1, leaving room for consideration of other word features and some subjectivity in the scoring. The four assumptions were that items that are dependent on prior SDA to recognize and correct in the SfV task, coded as 1, would have (1) relatively low spelling-to-pronunciation transparency of the target word; (2) relatively large phonological Levenshtein distance (PLD) between the stimulus and the target response (e.g., /tɑngu/ to tongue, coded as 1, requires 4 transformations between the mispronunciation and correct pronunciation vs /blɪnd/ to blind, coded as 0, requires only 1 transformation); (3) relatively high phonological-orthographic (P-O) surprisal, where *higher* values represent less likely pairings between a phoneme in the mispronunciation and its corresponding grapheme in the target word’s spelling (e.g., surprisal of / eɪʧ/ to ache, coded as 1, is 2.76, more than eight times the .34 surprisal of /blɪnd/ to blind, coded as 0); or (4) relatively low similarity between the target word and its nearest phonological neighbors (e.g., pigeon, coded as 1, requires an average of 2.4 transformations to create the nearest 20 phonological neighbors vs money, coded as 0, only requires 1 change to do so). Cohen’s kappa for the independent scoring is .95, indicating high inter-rater reliability. After independent scoring, the two scorers (coauthors of this paper) discussed and compared judgments based on the four relevant word features, agreeing on final scoring for all but one word, which was resolved by a third scorer based on one of the stated assumptions. Of the 40 words’ final coding, 19 words were coded as 0 and 21 words were coded as 1.

A series of word feature comparisons were checked between words coded as 0 and 1 on prior SDA dependence to explore features of words that were believed to be dependent on prior SDA to be recognized and corrected in the SfV task (see Table 2), including the four features that guided the scoring procedure. Compared to words coded as 0 on prior SDA dependence, words coded as 1 are significantly shorter in phonemes and syllables. All other comparisons were not significant between words coded as 0 and 1 on prior SDA dependence, although words coded as 0 tend to be longer in letters (*p* = .06), more transparent in spelling-to-pronunciation (*p* = .09), and shorter in target-stimulus PLD (*p* = .07) compared to words coded as 1. For more detailed by-item descriptive values for key features shown in Table 2, see Appendix B.

#### Frequency.

The dependent measure’s words’ log-transformed frequency values are based on the Hyperspace Analogue to Language (HAL) corpus taken from the English Lexicon Project (ELP; Balota et al., 2007). The list of target words’ log transformed HAL frequency ranges from 6.67 to 12.53.

#### Number of phonemes.

This is the number of phonemes in each target word, taken from the ELP (Balota et al., 2007) and ranging from 2 to 7 in the dependent measure.

#### Phonological Levenshtein distance 20.

PLD20 (Yap & Balota, 2009; Yarkoni et al., 2008) is the average number of transformations required through phoneme deletions, insertions, or substitutions (i.e., Levenshtein distance) to change a word into its 20 closest phonological neighbors in the ELP (Balota et al., 2007). The PLD20 values reported for the list of target words ranges from 1 to 2.75.

#### Spelling-to-pronunciation transparency rating.

To address the distance between a word’s correct pronunciation and its decoded form (i.e., how easily a word’s correct pronunciation can be derived from traditional orthographic-phonological relationships based on a word’s spelling), we used word ratings from Edwards et al., (2024) database. Raters were instructed to pretend that a letter string was unfamiliar to them, try applying a letter-to-sound reading strategy to the letter string, and rate the difficulty of matching a word’s decoded form to the word’s standard pronunciation (provided via audio recording for reference) on a Likert scale from 1 to 6 (1 = very easy to match/highly transparent, 6 = very difficult to match/highly opaque). Ratings from this database, including ratings for the 40 target words in this study, have been shown to be reliable based on their high correlation with expert ratings reported by Steacy et al. (2017). The range of ratings reported for the list of target words is 1.3 to 3.96.

### Data analytic procedures

A logistic regression was first conducted to determine how much of the variability in prior SDA dependence could be explained by word features represented by the four word features that guided the coding procedure and three other potentially relevant word features. Next, crossed random-effects models were used to predict participants’ SfV accuracy of a specific item (e.g., island) coded as a dichotomous response (1 = correct, 0 = incorrect) using child-level characteristics, general wordlevel characteristics, item-specific responses to other tasks, and an item-specific-by-word-level interaction as predictors (see Table 3 for examples of each predictor type). The models are “cross-classified” because SfV task responses originate from the intersection of each participant responding to each word in the same set of SfV items, where words are treated as a random factor that allows variability in SfV to be explained by word-level characteristics (e.g., frequency) and participants are treated as a random factor that allows variability in SfV accuracy to be explained by child-level characteristics (e.g., decoding fluency). Simulation studies from Cho et al. (2012) have demonstrated the robustness of these models to participant sample size and number of items, “resulting in more power than typical individual regression models” (Steacy, 2020, p. 156).

These models assume nonindependence in the data due to participants’ responses to the same set of words in the target SfV measure (i.e., nested responses within both child and word), which is accounted for by the inclusion of a by-subject random intercept and by-item random intercept in the models (see Brauer & Curtin, 2018). All reported analyses were conducted using a binomial distribution with a logit link, available through the glmer function in the lme4 package (Bates et al., 2015) from R programming (R Development Core Team, 2012). All continuous child and word predictors were grand mean-centered to aid in interpretation of the intercept and coefficients. The R code used to conduct the crossed-random effects models can be found in the supplementary files.

## Results

Descriptive statistics are provided in Table 4 for total target SfV accuracy, child-level, and word-level variables, along with zero-order correlations for child features. Point-biserial correlations are reported between prior SDA dependence and the other word-level variables, and zero-order correlations are reported between the remaining word-level variables. The bivariate dichotomous correlation between the items on the word reading and SfV tasks is .35.

Results of the logistic regression showed that 77.36% of the variance in prior SDA dependence could be collectively accounted for by the PLD between the target word’s correct pronunciation and its mispronunciation heard in the SfV task, the maximum P-O surprisal from the mispronunciation heard in the SfV task to the target word’s correct spelling, and the following features of the target words: PLD20, phoneme length, letter length, spelling-to-pronunciation transparency rating, and printed frequency (see Table 5). Given that the inter-rater reliability of the prior SDA dependence variable is .95, the maximum explainable variance is 95%, suggesting that approximately 13.57% of the reliable variance^4^ cannot be accounted for by word features typically associated with word reading difficulty. The remaining unexplained variance, therefore, likely reflects true construct-level distinctiveness between the prior SDA dependence variable and the other word features.

Separate analyses show that when only the four word features that informed the prior SDA dependence scoring procedure compete for variance in predicting item-level SfV performance, only 20.43% of the word variance is accounted for, and PLD20 emerges as the sole significant predictor (see Supplementary Table S1). When prior SDA dependence coding is added to the predictors, 16.58% additional variance in SfV performance is accounted for, and this variable becomes the sole significant predictor. Together, these results indicate that prior SDA dependence captures unique information about the items in the SfV task beyond the effects of word features that are correlated with and possibly contributing to this variable.

To decompose variance in SfV accuracy, we ran a series of crossed-random effects models (described in the data analytic procedures section above). First, we ran an unconditional model to determine how much variance was accounted for by person- and word-level predictors to compare with alternate models. The unconditional model’s intercept indicated that for an average child on an average SfV item, the probability of getting SfV items correct was .27 (see Table 6). This value is based on a conversion from the logit intercept of −1 to a probability, based on the following equation, where $p_{ji}$ represents the probability of a correct response from child j on item i and $-\gamma_{000}$ is the intercept: $p_{ji}=\frac{1}{1+\text{exp}(-\gamma_{000})}$. Variance estimates at the child level (1.08) and word level (1.09) suggested that there was significant variance to be explained at both levels in subsequent models.

A series of models (i.e., main effects and interaction) are presented in Table 6, illustrating separate sources of variance in item-level SfV accuracy associated with child-level, word-level, item-specific, and item-by-word predictors. All child-level, word-level, and item-specific predictors were entered into the main effects model simultaneously to predict SfV accuracy. All child-level predictors were significant, indicating that children were more likely to correctly identify a word from its mispronunciation in the SfV task if they had higher performance in phonological awareness, decoding, expressive vocabulary, and word reading. SDA dependence was the only significant word-level predictor of SfV accuracy, indicating that words coded as 0 (i.e., could be recognized from their mispronunciations and corrected without having successfully decoded the word before) were more likely to yield correct responses across the sample than words that depend on prior SDA. Item-specific word reading accuracy was also a significant predictor of SfV accuracy, indicating that a child was more likely to recognize the word and correctly respond to the SfV item if they could accurately read that word.

Next, we added the item-by-word interaction of item-specific word reading accuracy by prior SDA dependence to other predictors in the previous model (see Table 6). The same predictors from the previous model remained significant, and the new interaction was a significant predictor of SfV accuracy. Figure 1 shows how the ability to correctly read a given word significantly boosts a child’s probability of getting that word right in the SfV task, with much less noise around the probability estimates of words that are coded as 1 (i.e., dependent on prior SDA and generally more difficult items in the SfV task than words coded as 0) compared to the noise around probabilities of SfV accuracy for words coded as 0 (i.e., not dependent on prior SDA but clearly still impacted by prior reading experience). Also of note is the difference in likelihood of SfV accuracy based on prior SDA dependence when the word could not be read accurately: words that are not dependent on prior SDA have a ~30% chance of being recognized and corrected in the SfV task. But when the word is dependent on prior SDA, the chance of identifying the word from its mispronunciation drops to below 10%, meaning that even though the child is hearing a mispronunciation of a word that is likely in their oral vocabulary, the lack of prior experience with reading that word correctly makes it nearly impossible for the child to recognize the word from its mispronunciation.

### Alternate models

To consider other possible explanations for the effects of SDA dependence on SfV accuracy, we ran a set of alternate models investigating the contributions of other word-level features to item-level SfV accuracy (see Table 7). To provide alternate codes, dichotomous variables were created to code the 40 SfV words as 0 or 1 based on random coding (i.e., each word randomly coded as 0 or 1), mean split of spelling-to-pronunciation transparency rating (i.e., above or below the average transparency rating for the 40 words), and mean split of number of phonemes. In the subsequent interaction models, the SDA variable was replaced by each of the listed dichotomous word-level variables one at a time while maintaining the same word-level predictors from the interaction model in Table 6. First, we found that the 0/1 coding based on random assignment (i.e., each of the words in the SfV task randomly assigned to 0 or 1) was non-significant as a competing predictor, and it did not have a significant interaction with item-specific word reading accuracy. Next, we replaced the SDA variable with dichotomous coding based on the mean split of spelling-to-pronunciation transparency rating. There was a significant interaction of 0/1 coding based on mean split of transparency by item-specific word reading (see Figure 2). Words that were rated below the mean rating (i.e., more transparent from spelling to pronunciation, coded as 0) were approximately 10% more likely to be recognized and corrected in the SfV task than words above the mean rating (i.e., less transparent, coded as 1), both for words that were read correctly and incorrectly (see Table 7). While this interaction resembles the interaction of item-specific reading by dependence on prior SDA in Figure 1, the error bars around each of the estimates for words with lower transparency in Figure 2 are much larger than the error bars around each of the estimates for words coded as dependent on prior SDA in Figure 1, suggesting higher uncertainty of the transparency ratings’ effects on SfV performance compared to the effect of the SDA coding on SfV performance. In addition, a main effects model excluding item-specific word reading accuracy^5^ showed that only 26.24% of word-level variance was explained when the SDA variable was replaced by 0/1 coding based on mean split of transparency, compared to 38.14% explained with the SDA variable and the same other word-level predictors (see Supplementary Table S2), suggesting that dependence on prior SDA is capturing more unique information about word-level contributions to SfV accuracy.

Next, we found a significant main effect of 0/1 coding based on mean split of number of phonemes on SfV accuracy at *p* = .02 (see Supplementary Table S3), suggesting that dependence on prior SDA in the main effects model from Table 6 was likely competing for the same small amount of word-level variance as number of phonemes. This can be interpreted as dependence on prior SDA capturing the effect of length in phonemes on SfV accuracy. The interaction of the mean split of number of phonemes by item-specific word reading, however, was not a significant predictor of SfV accuracy. In the last two models (see Supplementary Table S3), we found non-significant main effects or interaction effects of 0/1 coding of words based on mean split of frequency or length in letters, suggesting that neither of these word features could explain the effects of dependence on prior SDA reported in Table 6, neither as competing predictors nor in an interaction with item-level word reading accuracy.

In addition, we created a dichotomous variable to represent whether the target word was within the nearest phonological neighbors of the mispronunciation in the SfV task (i.e., the stimulus). Using the phonological Levenshtein distance (PLD) between the target word and stimulus, a word was coded as 0 if the PLD is 1, as this indicates that the target is at least as close to the stimulus as the other nearest phonological neighbors that can be generated by only changing 1 phoneme. The word was coded as 1 if the PLD between the target and stimulus is >1, as this indicates that either (a) the target is not in the set of the nearest phonological neighbors that can be generated by only changing 1 phoneme or (b) the stimulus does not have any phonological neighbors that can be generated by only changing 1 phoneme. The results from this final model nearly replicate the interaction from Figure 1, suggesting that the effect of target-stimulus PLD on probability of SfV accuracy, moderated by item-specific word reading accuracy, is very similar to that of prior SDA dependence (see Table 7 and Figure 3), including similar stability of each probability estimate. The similarity of these findings prompted a closer analysis of the words that were coded as 1 for each variable to check for overlap between the two approaches to coding (see Figure 4). The chart shows that 13 of 21 words coded as being dependent on prior SDA have a target-stimulus PLD of >1. However, eight words (e.g., soup, deaf, veins) which have a target-stimulus PLD of 1, were also coded as being dependent on prior SDA. Similarly, a mix of words coded as being independent of prior SDA included words across the target-stimulus PLD range. Furthermore, a main effects model excluding item-specific word reading accuracy showed that only 30.65% of word-level variance was explained when the SDA variable was replaced by 0/1 coding based on the target-stimulus PLD, compared to 38.14% explained with the SDA variable and the same other word-level predictors (see Supplementary Table S2), suggesting that dependence on prior SDA is capturing more unique information about word-level contributions to SfV accuracy.

## Discussion

The current study’s goal was to explore the influence of prior word reading experience on item-level SfV performance by partitioning variance in task performance into child-level, word-level, item-specific, and item-by-word features. Our interest in examining the influence of item-level reading ability on set for variability (SfV) performance stems, in part, from the low accuracy rates reported by Edwards et al. (2022) on the SfV task. This prompted us to consider whether item-level reading ability might affect SfV performance, particularly for items with larger phonological distances between the output of decoding and the stored lexical pronunciation. Theoretically, we hypothesized that some of the SfV items must first be decoded before they can be successfully disambiguated within the SfV task. This hypothesis is grounded in the lexical quality hypothesis (Perfetti, 1992, 2017; Perfetti & Hart, 2002). Specifically, high-quality lexical representations in the orthographic lexicon include redundant phonological forms – “one from spoken language and one recoverable from orthographic-to-phonological mappings” of the word (Perfetti & Hart, 2002, p. 190). This suggests that performance on certain SfV items may be influenced by whether a child has previously decoded the word and incorporated the decoded form into its stored representation.

Below we examine properties of words we coded as dependent on prior SDA (successful decoding attempts) in order to recognize and correct in SfV task. Next, we discuss our interpretation of results from testing the probability of successfully completing the SfV task conditionalized on item reading accuracy and whether the word was considered to require prior SDA. Last, we highlight the alternate model results that pose other potential word-level explanations of the item-by-word interaction result, followed by implications for future empirical investigations of SfV performance and SfV assessment in practice.

When we examined average differences in word features between words coded as 0 and 1 on prior SDA dependence, we found that words coded as 0 were significantly longer in phonemes and syllables. This suggests that words with more phonological information available during the SfV task may allow more opportunities for disambiguating the mispronunciation and arriving at the correct pronunciation without having had prior SDA with these words. Words coded as 0 were also longer in letters, more transparent in spelling-to-pronunciation, and shorter in phonological Levenshtein distance (PLD) between the target word and stimulus (i.e., mispronunciation in the SfV task). Although these differences were not statistically significant, we acknowledge that in our limited sample size of 40 words, we cannot establish the absence of an effect from these variables on the grouping of words based on the prior SDA coding variable, as shown by non-significant results of post hoc equivalence tests. While the initial coding of the SfV items was informed by the assumption that words with a higher degree of irregularity (i.e., less transparent) may be more dependent on a prior SDA than more transparent words, the restricted range of higher spelling-to-pronunciation transparency (1.3–3.96 on a scale of 1–6, where 1 = highly transparent, 6 = highly opaque) represented in the 40 SfV task items may partially explain the non-significant difference in transparency between words coded as 1 versus 0; none of the words had very low transparency scores, nor did prior SDA correlate with transparency ratings. Therefore, it is possible that in a different sample of items selected to represent a wider range of spelling-to-pronunciation transparency, dependence on prior SDA may be more highly correlated with transparency of words. Additionally, words did not significantly differ on PLD20 of the correct response or P-O surprisal of the mispronunciation to target word’s spelling, suggesting that while these features were thought to be influential during the prior SDA coding of the SfV task items, there was no systematic pattern of these features that could distinguish words coded as dependent on, versus independent of, prior SDA.

Beyond these direct comparisons, the logistic regression model shows that 77.36% of the variance in prior SDA dependence could be collectively accounted for by the target-stimulus PLD, the maximum P-O surprisal from the mispronunciation heard in the SfV task to the target word’s spelling, and the target words’ PLD20, phoneme length, letter length, spelling-to-pronunciation transparency rating, and printed word frequency. Furthermore, item-level analyses reveal that when only the word features from the prior SDA dependence scoring procedure compete to predict SfV performance, PLD20 emerges as the sole significant predictor. When prior SDA dependence is added, it becomes the sole significant predictor and accounts for substantially more variance, strengthening the interpretation that this variable captures unique information about the SfV task items beyond the effects of word features that were thought to influence the distance between a word’s decoded and the lexical pronunciations.

The Main Effects Model results support the notion that SfV accuracy on some words, but not all, is dependent on having had prior SDA with those specific words, as represented by the significant contribution of prior SDA dependence to SfV performance. Words coded as 0 (i.e., could be recognized from their mispronunciations and corrected without having successfully decoded the word before) were more likely to yield correct responses across the sample than words coded as 1 (i.e., dependent on prior SDA). Together with the significant contribution of item-specific word reading accuracy to SfV accuracy, these results indicate that while being able to read a word correctly helps to recognize it from its mispronunciation in the SfV task, prior SDA is only essential for some words in the SfV task in order to recognize them and provide the correct pronunciation, and the ability to read the word correctly still does not guarantee SfV accuracy. This finding of prior SDA as necessary but not sufficient for a subset of words in the SfV task is consistent with the notion that phonological distance between the output of decoding and the stored lexical pronunciation varies as a function of not only a child’s knowledge and reading experiences, but also word features that impact spelling-sound relationships (see Edwards et al., 2022; Siegelman et al., 2020; Steacy et al., 2022).

The significant interaction of item-specific word reading accuracy by SDA coding in the subsequent model also supported our assumption that while some words can be recognized in the SfV task without having a prior SDA, there are some words that would be nearly impossible for a child to recognize from their mispronunciations if they lack prior experience with successfully reading those words. Given the average child’s base rate of a 27% probability of providing a correct response on the SfV task in this sample, we expected to see that for words coded as 0, a child would have a similar probability of recognizing and correcting it in the SfV task without being able to read that word correctly, which is the case (see Figure 1). Their probability of SfV accuracy increases to about 50% if they can read the word correctly, but the larger error bars for words coded as 0 suggest there is some variability in what we could predict for SfV accuracy of words that we do not consider to be dependent on prior SDA to recognize in the SfV task. There is likely more room for individual differences in child-level skills, like general decoding, phonological awareness, vocabulary, and word reading, as well as item-specific vocabulary knowledge, to influence a child’s approach to these items in the SfV task (see Edwards et al., 2022; Siegelman et al., 2020; Steacy et al., 2022; Wegener et al., 2022). However, for words coded as 1, we find that the average probability of getting a correct response for these words, without being able to read it correctly, is less than 10%, far below the 30% probability of SfV accuracy for words coded as 0. The error bar is much smaller for this set of circumstances, suggesting that the fixed properties of the items themselves are likely driving the difficulty in the SfV task if the child hasn’t had a prior SDA with these items before. For words read correctly and coded as 1, the ~25% probability of correctly recognizing these in the SfV task is also quite stable, suggesting that the prior experience with decoding these words is likely influencing the child’s approach to responding to these words in the SfV task. This is consistent with Steacy et al., (2019) suggestion that children may apply at least two different approaches to the SfV task, both of them relying on existing orthographic representations of the particular items being tested. The first strategy is accessing the redundant phonological representation that was stored from the prior SDA with a given word (Elbro & de Jong, 2017; Steacy et al., 2023). As children gain more experience in reading and spelling, they may apply a second strategy by actively translating the mispronunciation to spelling using general knowledge of English orthography, and then accessing the correct phonological representation(s) associated with the generated orthographic string stored in memory (i.e., orthographic facilitation). For a child who can read a word that is dependent on prior SDA, both of the strategies should lead to success in the SfV task, whereas neither of these strategies would be helpful if the child has never encountered the word in text before, hence the stabler probability estimates for words that are dependent on prior SDA. For words that are not dependent on prior SDA to recognize in the SfV task, the less stable estimates likely reflect items in the task that are more demanding on a child’s global phonological system and less demanding of specific orthographic representations available in a child’s lexicon.

Given the subjective nature of the prior SDA dependence scoring procedure, we ran a set of alternate models investigating the contributions of other word-level features to item-level SfV accuracy to consider other possible explanations for the effects of prior SDA dependence on SfV accuracy. Overall, the first model based on randomized dichotomous coding suggests that the results of prior SDA dependence were not merely by chance, but rather, meaningful information about the items was captured by the prior SDA dependence variable. When we replaced prior SDA dependence with dichotomous coding based on the mean split of spelling-to-pronunciation transparency rating, the significant interaction of transparency by item-specific word reading in Figure 2 revealed that more transparent words were approximately 10% more likely to be recognized and corrected in the SfV task than less transparent words, regardless of whether a child could read the word correctly. While this interaction resembles the interaction of item-specific reading by dependence on prior SDA in Figure 1, the error bars around each of the estimates are larger, suggesting higher uncertainty of transparency effects on SfV performance. In addition, 15% more unique word-level variance in SfV accuracy was accounted for in a model with prior SDA dependence compared to an alternate model that replaced prior SDA dependence with the mean split of transparency variable; together with the non-significant difference in transparency rating between words coded as 0 and 1 in prior SDA dependence, we believe that the prior SDA dependence variable captures information about words that require a bigger jump from the pronunciation generated in a decoding attempt to a correct pronunciation, beyond what might be explained by a word’s spelling-to-pronunciation transparency.

Next, we replaced prior SDA dependence with dichotomous coding based on whether the target was within the stimulus’ nearest phonological neighbors. The significant interaction of this variable by item-specific word reading in Figure 3 showed that SfV accuracy is impacted by a child’s ability to read the word correctly, regardless of the target-stimulus PLD, but if the target is within the nearest phonological neighbors of the stimulus, children have a higher likelihood of recognizing the word in the task, and this effect is stable across children who can and cannot read the word correctly. On the surface, this finding could appear to reduce the effect of the novel prior SDA dependence variable to another more objective feature of the items, and it indeed provides useful information about why some items on the SfV task are more difficult than others. However, upon closer examination of the two variables in Figure 4, we found that of the 19 words determined to not be dependent on prior SDA, 12 of them were within the nearest phonological neighbors of their mispronunciations in the SfV task. The other seven words were spread across the range of PLD between target and stimulus, showing that again, there is not a systematic pattern of another word feature that can fully account for the prior SDA dependence coding. Our claim that prior SDA dependence represents a unique property of the words is further strengthened by the weak non-significant point-biserial correlation of .29 between prior SDA dependence and target-stimulus PLD, despite this feature being identified as a possible influence on prior SDA dependence the coding procedure. The remaining alternate models confirmed that the significant interaction of item-specific word reading accuracy by prior SDA dependence could not be accounted for by interactions of item-specific word reading and mean split based on number of phonemes, frequency, or letter length.

Overall, our findings suggest that for some SfV items, prior successful decoding experience may be necessary for accurate mispronunciation correction; this implies that how SfV items are selected can affect the relationships between the task and word reading skill. Words coded as 0 are likely better candidates for creating a measure of SfV that captures general ability in mispronunciation correction that is not dependent on having had prior SDA with specific items in order to complete the task. Theoretically, this means that there are words for which a child’s responses could reveal more about the efficiency of the phonological cleanup mechanism in the reading system. Words coded as 1, however, are likely better candidates for creating a measure of SfV that reflects how prior decoding experience with specific items has contributed to building high quality representations of those specific items. Future studies of SfV in relation to different cognitive or linguistic skills might consider comparing the value of (a) knowing how general SfV ability relates to general performance on an outcome of interest (e.g., word reading, spelling, vocabulary) when competing with other child-level predictors to (b) knowing how different word-level, child-level, and item-specific predictors account for variation in quality of item-specific representations. As noted above, the 40 words in the current study’s SfV task represented a restricted range of spelling-to-pronunciation transparency (1.3–3.96), with none of the words having transparency ratings of 4–6, which would represent higher difficulty in arriving at the correct pronunciation of a work based on its spelling (i.e., lower transparency). Future analyses of SfV might explore effects of prior SDA dependence on SfV accuracy when items with lower transparency are included in the task. Given the high inter-rater reliability of the prior SDA dependence coding, we predict that other working groups (with at least two scorers who have detailed knowledge of English phonics and decoding rules) can account for the same relevant criteria from our scoring procedure described earlier and therefore, reliably code new words for their dependence on prior SDA in future projects.

Moreover, the current study did not address the finer grained influence of the number of additional plausible pronunciations for each item in the SfV task, nor did we distinguish between items that could have multiple plausible pronunciations based on smaller versus larger grain size units. For example, the mispronunciation of measles in the current study’s task is just one of multiple ways that the word could be decoded based on variation of the *ea* and *s* graphemes, while mispronunciations of pudding would likely be more restricted to variation in the *u* grapheme. It is possible that the prior SDA dependence variable is capturing some of this effect of variant vowels, but it remains unclear if words with more plausible mispronunciations would vary in their dependence on prior SDA to disambiguate in the SfV task, moving beyond the dichotomous prior SDA dependence variable presented here. Future studies could investigate the relationship between number of plausible mispronunciations at different grain sizes (i.e., decoding single graphemes versus syllables) and prior SDA dependence, as well as how these two variables compete for variance in item-level SfV accuracy.

This work also has implications for how SfV might be conceptualized as a potentially useful tool in predicting risk for reading difficulties. Steacy et al. (2023) found that the strength of the relationship between SfV and word reading was higher at the lower end of reading for English-speaking students in grades 2–5, suggesting that “the SfV task in the bottom end of word reading distribution may represent a pure measure of phonological cleanup that is less contaminated by the presence of stored spelling pronunciations and feedback from stored spelling representations” (p. 265). It is important to highlight that this finding was drawn from children’s performance on a SfV task of 75 items, including the 40 items in the current study’s SfV task, meaning that the task included a blend of words that would and would not be dependent on prior SDA to recognize in the task. Given our earlier distinction of some items being better candidates for creating a SfV measure that captures general child-level ability in mispronunciation correction (i.e., words coded as 0) versus better candidates for capturing information about item-specific decoding experience (i.e., coded as 1), we propose a caveat to Steacy et al.’s suggestion about SfV’s potential as a powerful early predictor of dyslexia risk: creating a SfV measure of general ability for early prediction purposes should involve selection of items that are not dependent on prior SDA to recognize in the SfV task, starting with words that are restricted in degree of irregularity (as determined by spelling-to-pronunciation transparency or PLD between the word’s correct pronunciation and its decoded form, for example). Conversely, a SfV task composed of items that are dependent on prior SDA and aligned with a scope and sequence of explicitly taught grapheme-phoneme correspondences might be more useful for tracking changes to quality of specific item representations, perhaps in the form of a progress monitoring tool to be used alongside other word reading and spelling progress monitors. Beyond these recommendations, we are left with more work to determine whether other word features and measures of item-specific knowledge (e.g., vocabulary, oral familiarity) not considered in the present study could guide the selection of items that allow measures of SfV ability to be useful tools in educational practice.

## Notes

1. For each phoneme in the mispronunciation, a conditional probability of the corresponding grapheme was calculated using the Kearns (2020) phonological database. Probability estimates were transformed into maximum surprisal values (i.e., −log(orthography ∣ phonology)), where higher values represent more unlikely pairings (see Siegelman et al., 2020 for more information).
2. The task consisted of 75 items administered to participants, but given that item-level word reading data was only collected for 40 of the 75 SfV items, only the responses to 40 of the SfV items are analyzed in the current study.
3. Given the constraints of how many word-level predictors can be entered into a single model before encountering issues with convergence, we prioritized modeling the effects of the prior SDA dependence variable and four competing predictors: one of the features thought to be most theoretically relevant to prior decoding experience (spelling-to-pronunciation transparency), the feature that accounted for the most word-level variance as a single predictor of SfV accuracy (PLD20), and two control variables (frequency and phoneme length). The other two word features (target-stimulus PLD and maximum P-O surprisal) were not included as predictors in the final models of SfV performance, but they were considered in the logistic regression model predicting prior SDA dependence from various competing word features.
4. This value was calculated by dividing .7736 (McFadden’s *R* from the logistic regression) by .95 (Cohen’s kappa for the prior SDA scoring), and subtracting the resulting value (.8143) from .95 to determine the unexplainable variance (13.57%) in the logistic regression model.
5. In order to accurately interpret the separate child- and word-level variance estimates of SfV accuracy in the same model, item-specific predictors are removed, as they can account for both child- and word-level variance when left in the model. See LaHuis et al. (2014) for further discussion.

## Supplementary Material

Supp 1

Supplemental data for this article can be accessed online at https://doi.org/10.1080/10888438.2026.2704734

## Figures and Tables

**Figure 1.**
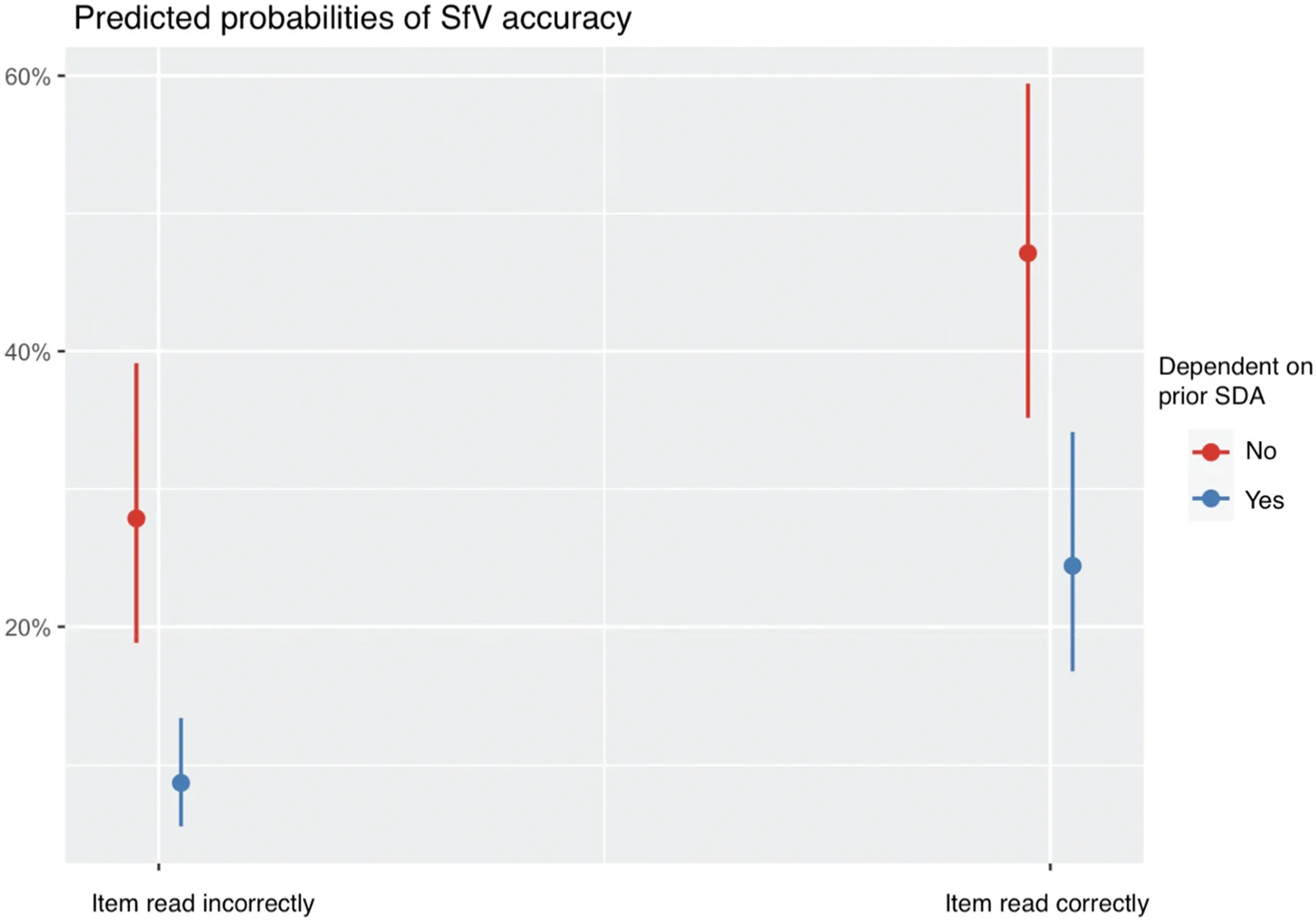
Interaction of item-specific word reading accuracy by dependence on prior successful decoding attempts (SDA) in predicting likelihood of correct responses in the set for variability (SfV) task. *Note*. The y-axis represents probability in percentage of having a correct response on an item in the SfV task. The x-axis represents whether the item from the SfV task was read correctly in the word reading task. In the legend, dependence on prior SDA refers to whether a word was coded as being dependent or not on prior successful decoding attempts to recognize in the SfV task.

**Figure 2.**
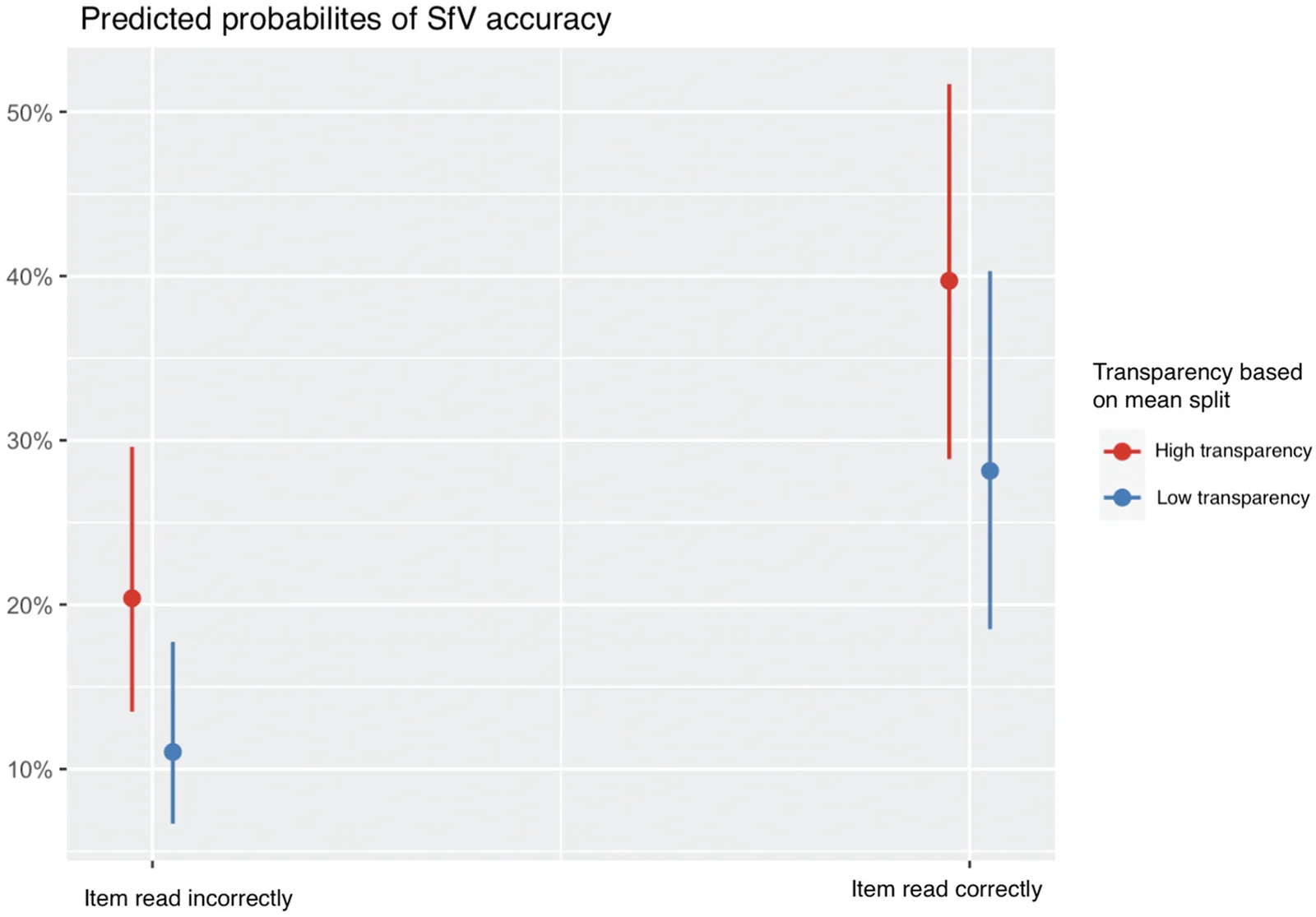
Interaction of item-specific word reading accuracy by mean split of spelling-to-pronunciation transparency in likelihood of correct responses on the set for variability (SfV) task. *Note*. The y-axis represents probability in percentage of having a correct response on an item in the SfV task. The x-axis represents whether the item from the SfV task was read correctly in the word reading task.

**Figure 3.**
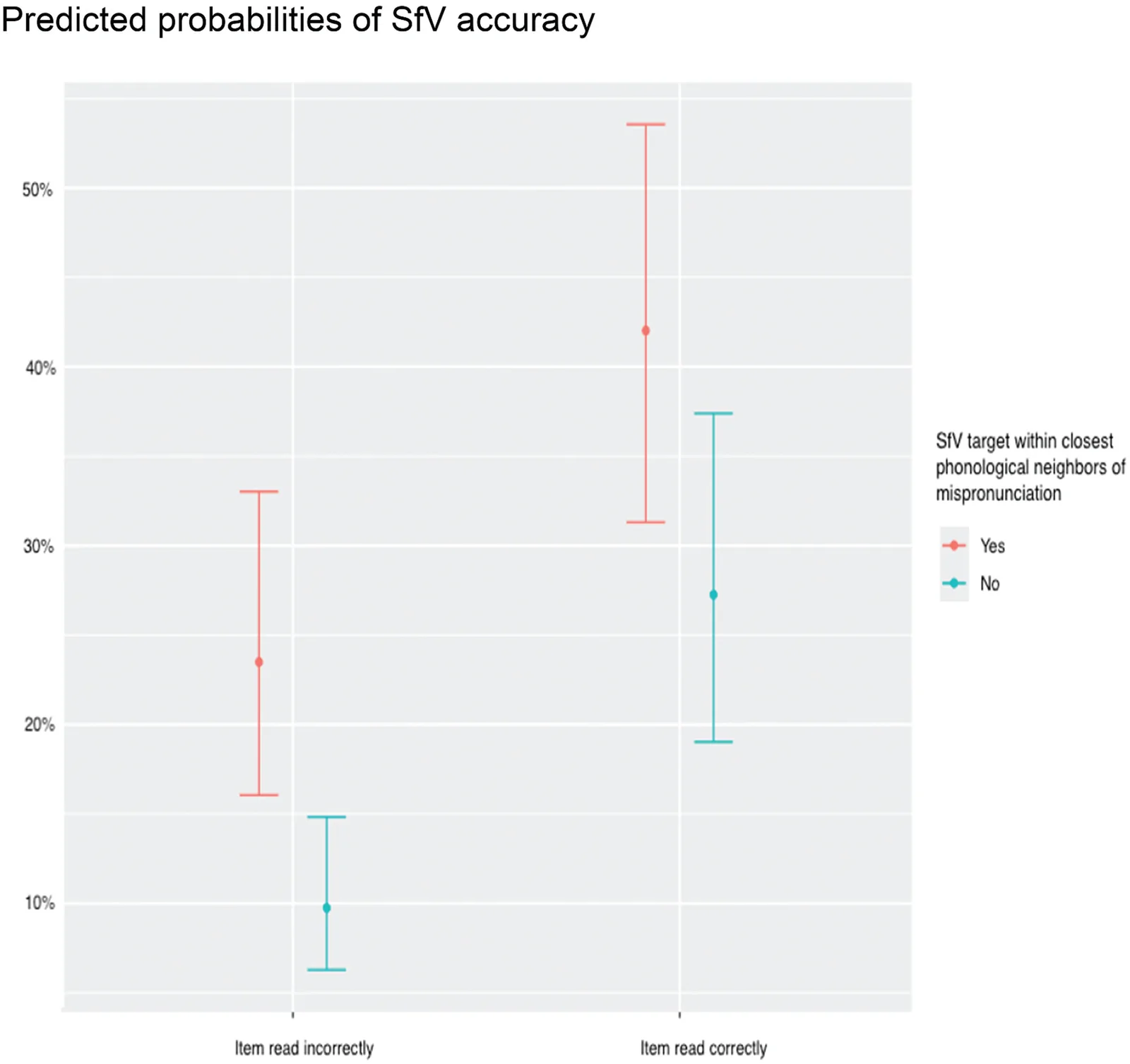
Interaction of item-specific word reading accuracy by coding of mispronunciations’ nearest phonological neighbors predicting the likelihood of correct responses in the set for variability (SfV) task. *Note*. The y-axis represents probability in percentage of having a correct response on an item in the SfV task. The x-axis represents whether the target word from the SfV task was read correctly in the word reading task. In the legend, red represents items (i.e., the target words) that were within the nearest phonological neighbors of the corresponding mispronunciations (i.e., the stimuli) in the SfV task.

**Figure 4.**
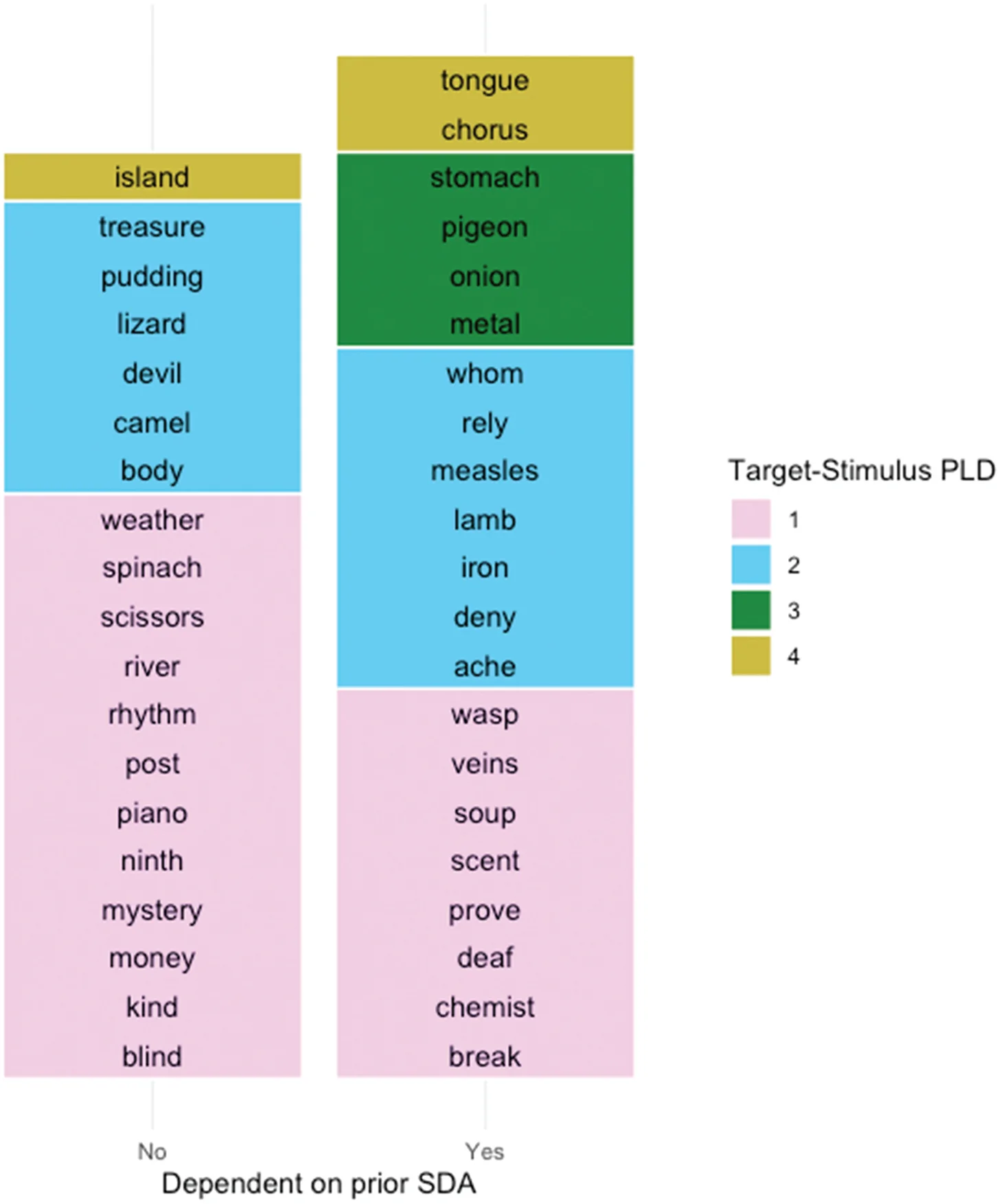
Set for variability task items (*N* = 40) plotted by the target-stimulus phonological Levenshtein distance (PLD) and dependence on prior successful decoding attempts (SDA).

**Table 1.** Demographic characteristics of participants (*N* = 499)

Variable	
Female	51.30%
English Language Learners	11.22%
Race/Ethnicity	
African American	40.08%
Hispanic	14.63%
White	41.08%
Asian	1.40%
Multiracial	2.00%
American Indian/Alaskan Native	0.20%
Native Hawaiian/Pacific Islander	0.40%
Age (Years)	9.16 (1.13)
Letter-word identification SS	101.53 (13.16)
Phonological awareness SS	8.74 (2.91)
Expressive vocabulary SS	10.12 (3.17)

Note. For age and standard scores (SS), mean values are presented, followed by standard deviations in the parentheses.

**Table 2.** Word feature comparison by dependence on prior successful decoding attempts (SDA) (*N* = 40).

Word features	SDA = 1 (*n* = 21)		SDA = 0 (*n* = 19)		*F* (1, 40)	*p*
	*M*	*SD*	*M*	*SD*		
Concreteness	3.94	1.10	4.17	0.87	.49	0.49
Letter length	5.00	1.07	5.74	1.25	3.83	0.06
Log HAL frequency	8.59	1.28	9.39	1.71	2.67	0.11
OLD20	1.93	0.38	2.07	0.56	.89	0.35
Phoneme length	4.00	1.02	4.68	0.80	5.20	0.03
Phonographic neighbors	0.62	1.05	1.32	1.92	1.98	0.17
P-O surprisal	2.64	2.33	3.79	3.61	1.38	0.25
PLD20	1.55	0.47	1.75	0.41	1.86	0.18
Semantic diversity	1.60	0.35	1.64	0.29	0.18	0.68
Syllable length	1.43	0.49	1.89	0.55	7.54	0.01
Target-Stimulus PLD	2.00	1.00	1.47	0.77	3.42	0.07
Transparency	2.76	0.63	2.37	0.75	3.04	0.09

Note. *M* and *SD* are used to represent mean and standard deviation, respectively.

HAL = hyperspace analogue to language; OLD20 = average orthographic Levenshtein distance to 20 closest orthographic neighbors; P-O = phonological-orthographic; PLD20 = average phonological Levenshtein distance to 20 closest phonological neighbors; Target-stimulus PLD = phonological Levenshtein distance between the target word and the stimulus (the mispronunciation) in the set for variability task; Transparency = spelling-to-pronunciation transparency rating.

**Table 3.** Types of measures used in analytic models.

Measure Type	Description	Example
Child-level	A participant’s total score on a given task	A participant’s raw total score on the Woodcock–Johnson III word identification task (a number between 0 and 76, based on how many words were correctly read by the individual participant).
Word-level	A fixed property of a word in the dependent measure	The number of phonemes in a specific word in the dependent set for variability (SfV) measure, such as island, which has five phonemes.
Item-specific	A participant’s individual response to a single word within a child-level measure that is also in the dependent measure	A participant’s word reading accuracy of a specific word (e.g., island, 0 = incorrectly read, 1 = correctly read) which also appears in the dependent SfV measure
Item-specific-by-word-level interaction	The interaction of a participant’s individual response to a single word within a child-level measure that is also in the dependent measure with a fixed property of that same word	The interaction of a participant’s word reading accuracy (0 or 1) of the word island and the log-transformed printed word frequency of island (10.71)

**Table 4.** Descriptive statistics and zero-order correlations of child (*N* = 499) and word (*N* = 40) features.

Child-Level Variable	*M*	*SD*	1	2	3	4		
1. Set for variability	27.00	11.25	–	–	–	–		
2. PDE	24.17	11.69	.73**	–	–	–		
3. Vocabulary	23.91	6.39	.51**	.37**	–	–		
4. PA	22.32	6.66	.66**	.61**	.48**	–		
5. Word reading	46.28	8.12	.80**	.78**	.47**	.61**		
Word-Level Variable	*M*	*SD*	1	2	3	4	5	6
1. Prior SDA dependence	–	–	–	–	–	–		
2. Log HAL frequency	8.97	1.57	−.26	–	–	–		
3. Phoneme length	4.32	1.00	−.35*	−.19	–	–		
4. PLD20	1.65	.46	−.22	−.40**	.75**	–		
5. Transparency	2.58	0.73	.27	−.49**	.26	.25	–	
6. Target-stimulus PLD	1.75	.93	.29	−.02	.01	−.02	.31	–
7. P-O surprisal	3.19	3.10	−.19	.01	.07	−.06	.36*	.06

*Note. M* and *SD* are used to represent mean and standard deviation, respectively.

** Correlations that were significant at *p* < .01.

* Correlations that were significant at *p* < .05. PDE = Phonemic Decoding Efficiency subtest from the TOWRE-2; PA = Phonemic Awareness subtest from the CTOPP; SDA = Successful Decoding Attempts; HAL = Hyperspace Analogue to Language corpus; PLD20 = average Phonological Levenshtein Distance to 20 closest phonological neighbors; Transparency = Spelling-to-Pronunciation Transparency Rating; Target-stimulus PLD = Phonological Levenshtein distance between the target word and the stimulus (the mispronunciation) in the set for variability task; P-O = Phonological-orthographic. Point-biserial correlations are reported for correlations between prior SDA dependence and the other six word-level variables, while zero-order correlations are reported for correlations between the remaining word-level variables.

**Table 5.** Logistic regression of prior successful decoding attempts (SDA) dependence coding variable.

Fixed effects	Est.	*SE*	*z*	*p*
**Intercept**	4.41	2.30	1.92	.06
**Word-Level Factors^a^**				
Letter length	−2.99	1.80	−1.66	.10
Log HAL frequency	**−3.64**	**1.84**	**−1.97**	**.049**
P-O surprisal	**−1.66**	**.73**	**−2.28**	**.02**
Phoneme length	.39	.63	−1.17	.24
PLD20	**−16.52**	**8.06**	**−2.05**	**.04**
Target-Stimulus PLD	1.31	1.13	1.16	.25
Transparency	**11.57**	**5.23**	**2.21**	**.03**
McFadden *R***^2^**		.77		

Note. Est.= parameter estimate; *SE* = standard error; HAL = hyperspace analogue to language; P-O = phonological-orthographic; PLD20 = average phonological Levenshtein distance to 20 closest phonological neighbors; Target-stimulus PLD = phonological Levenshtein pistance between the target word and the stimulus (the mispronunciation) in the set for variability task; Transparency = spelling-to-pronunciation transparency rating.

Each of the predictors and respective estimates represent the results from predicting the probability of a word being coded as 0 or 1 (not dependent versus dependent on prior successful decoding attempts to recognize and correct in the set for variability task) from all features of the target words in the task simultaneously (i.e., in the presence of all other word-level predictors in the model). aWordlevel factors represent fixed characteristics of the 40 words that remain constant across individualparticipants in the set for variability task.

**Table 6.** Fixed effects predicting probability of correct responses in the set for variability (SfV) task.

Fixed effects	Unconditional Model				Main Effects Model				Interaction Model			
	Est.	*SE*	*z*	*p*	Est.	*SE*	*z*	*p*	Est.	*SE*	*z*	*p*
**Intercept**	**−1.00**	**.23**	**−4.43**	**<.01**	**−1.12**	**.26**	**−4.29**	**<.01**	**−.95**	**.26**	**−3.67**	**<.01**
**Item-by-word Interactions** ^a^											
Word Reading × SDA	–	–	–	–	–	–	–	–	**.38**	**.10**	**3.87**	**<.01**
**Item-specific Factor** ^b^											
Word Reading	–	–	–	–	**1.03**	**.03**	**19.06**	**<.01**	**.83**	**.07**	**11.15**	**<.01**
Child-Level Factors^c^												
PDE	–	–	–	–	**.02**	**<.01**	**4.06**	**<.01**	**0.2**	**<.01**	**4.01**	**<.01**
PA	–	–	–	–	**.04**	**.01**	**6.35**	**<.01**	**.04**	**.01**	**6.44**	**<.01**
Vocabulary	–	–	–	–	**.02**	**.01**	**4.69**	**<.01**	**.02**	**.01**	**4.68**	**<.01**
Word Reading					**.04**	**.01**	**5.86**	**<.01**	**.04**	**.01**	**6.01**	**<.01**
**Word-Level Factors** ^d^												
Log HAL Frequency	–	–	–	–	.02	.13	.13	.90	.02	.13	.13	.90
N Phonemes	–	–	–	–	.24	.27	1.08	.28	.30	.27	1.11	.27
PLD20	–	–	–	–	.23	.59	.39	.69	.21	.58	.37	.71
SDA Coding	–	–	–	–	**−1.11**	**.38**	**−2.92**	**<.01**	**−1.39**	**.38**	**−3.64**	**<.01**
Transparency	–	–	–	–	.11	.27	.39	.70	.09	.27	.35	.73
Intercepts		Variance				Variance				Variance		
Child		1.08				0.22				.22		
Word		1.92				1.01				.98		

Note. Est.= parameter estimate; *SE* = standard error; SDA = successful decoding attempt; PDE = phonemic decoding efficiency subtest from the TOWRE-2; PA = phonological awareness; HAL = Hyperspace Analogue to Language corpus; PLD20 = average phonological Levenshtein distance to 20 closest phonological neighbors; Transparency = spelling-to-pronunciation transparency rating.

Each of the predictors and respective estimates represent the results from predicting the probability of word spelling accuracy from all variables simultaneously (i.e., in the presence of all other word- and person-level predictors in the model).

a An item-by-word interaction represents an interaction of an item-specific response’s accuracy (varying by participant and item in the task) by a feature of a word that remains constant across all participants.

b Item-specific factors represent item-specific perfor- mance;

c Child-level factors represent aggregate performance by the child on the measure.

d Word-level factors represent fixed characteristics of the 40 words that remain constant across individual participants in the set for variability task.

**Table 7.** Alternate fixed effects predicting probability of correct responses in the set for variability (SfV) task.

Fixed effects	Random Model				Mean Split Transparency Model^e^				Target-Stimulus PLD Model			
	Est.	*SE*	*z*	*p*	Est.	*SE*	*z*	*p*	Est.	*SE*	*z*	*p*
**Intercept**	**−1.42**	**.25**	**−5.75**	**<.01**	**−1.36**	**.25**	**−5.38**	**<.01**	**−1.19**	**.24**	**−4.92**	**<.01**
**Item-by-word Interactions** ^a^												
Word Reading ×Alternate Feature*	−.01	.10	−.05	.96	**.20**	**.10**	**2.08**	**.04**	**.38**	**.10**	**3.96**	**<.01**
**Item-specific Factor** ^b^												
Word Reading	**1.04**	**.07**	**15.22**	**<.01**	**.94**	**.07**	**13.18**	**<.01**	**.86**	**.07**	**12.33**	**<.01**
**Child-Level Factors** ^c^												
PDE	**.02**	**<.01**	**4.06**	**<.01**	**.02**	**<.01**	**4.03**	**<.01**	**.02**	**<.01**	**4.05**	**<.01**
PA	**.04**	**01**	**6.35**	**<.01**	**.04**	**.01**	**6.37**	**<.01**	**.04**	**.01**	**6.36**	**<.01**
Vocabulary	**.02**	**.01**	**4.69**	**<.01**	**.02**	**.01**	**4.67**	**<.01**	**.02**	**.01**	4.67	**<.01**
Word Reading	**.04**	**.01**	**5.85**	**<.01**	**.04**	**.01**	**5.88**	**<.01**	**.04**	**.01**	**5.84**	**<.01**
**Word-Level Factors** ^d^												
Alternate Feature	−.58	.37	1.58	.11	−.72	.40	−1.81	.07	**−1.05**	**.34**	**−3.04**	**<.01**
Log HAL Frequency	.04	.14	.25	.80	.03	.14	.21	.84	.08	.13	.62	.53
*N* Phonemes	.52	.28	1.89	.06	**.58**	**.27**	**2.10**	**.04**	.43	.27	1.61	.11
PLD20	.21	.63	.33	.74	.15	.64	.23	.82	.39	.60	.65	.52
Transparency	−.10	.28	−.35	.73	–	–	–	–	−.08	.27	−.28	.78
Intercepts		Varience				Varience				Varience		
Child		.24				.22				.22		
Word		1.14				1.17				1.07		

Note. Est. = parameter estimate; *SE* = standard error; PLD = phonological Levenshtein distance; PDE = phonemic decoding efficiency; PA = phonological awareness; HAL = Hyperspace Analogue to Language; PLD20 = average phonological Levenshtein distance to 20 closest phonological neighbors; Transparency = spelling-to-pronunciation transparency rating. Each of the predictors and respective estimates represent the results from predicting probability of word spelling accuracy from all variables simultaneously (i.e., in the presence of all other word- and person-level predictors in the model).

a An item-by-word interaction represents an interaction of an item-specific response’s accuracy (varying by participant and item in the task) by a feature of a word that remains constant across all participants.

b Item-specific factors represent item-specific performance;

c Child-level factors represent aggregate performance by the child on the measure.

d Word-level factors represent fixed characteristics of the 40 words that remain constant across individual participants in the SfV task.

e The mean split transparency model excluded the transparency rating from the predictors in the model as it would compete with the mean split transparency variable for the same variance.

* The alternate feature varies depending on the model: in the Random Model, the alternate feature is the coding of each word in the SfV task based on random assignment to 0 or 1. In the Mean Split Transparency Model, the alternate feature is the dichotomous coding of each word based on whether its spelling-to-pronunciation falls below or above the mean transparency rating of the 40 words in the SfV task (0 vs 1, respectively). In the Target Stimulus-PLD model, the alternate feature is a dichotomous variable representing whether the target word was within the nearest phonological neighbors of the mispronunciation in the SfV task (i.e., the stimulus) or not (0 vs 1, respectively).

## Appendices

### Appendix A

**Table:**

Word	Mispronunciation (phonetic transcription)	Dependent on prior successful decoding attempt (SDA)	Mean split of spelling-to-pronunciation transparency rating	Target- Stimulus PLD
ache	eɪʧ	1	1	2
blind	blɪnd	0	0	1
body	boʊdi	0	0	2
break	brik	1	0	1
camel	keɪmɛl	0	0	2
chemist	ʧɛmɪst	1	1	1
chorus	ʧɔrʊs	1	1	4
deaf	dif	1	0	1
deny	dɛni	1	0	2
devil	divɪl	0	0	2
iron	aɪrɑn	1	0	2
island	ɪzlɪnd	0	1	4
kind	kɪnd	0	0	1
lamb	læmbə	1	0	2
lizard	lɪzɑrd	0	0	2
measles	misəls	1	1	2
metal	mitæl	1	0	3
money	moʊni	0	0	1
mystery	maɪst i	0	0	1
ninth	nɪnɵ	0	1	1
onion	ɑniɑn	1	1	3
piano	paɪænoʊ	0	0	1
pigeon	pɪgiɑn	1	1	3
post	pɑst	0	0	1
prove	proʊv	1	0	1
pudding	pədɪŋ	0	0	2
rely	rɛli	1	0	2
rhythm	raɪðəm	0	1	1
river	raɪv	0	0	1
scent	skɛnt	1	1	1
scissors	skɪz z	0	1	1
soup	saʊp	1	1	1
spinach	spɪnæʧ	0	1	1
stomach	stoʊmæʧ	1	1	3
tongue	tɑngu	1	1	4
treasure	ʧri	0	0	2
veins	vinz	1	1	1
wasp	wæsp	1	0	1
weather	wið	0	1	1
whom	wɑm	1	1	2

Note. 1 = dependent on prior SDA, 0 = not dependent on prior SDA.

Spelling-to-pronunciation transparency ratings from Edwards et al. (2024) database represent how easily a word’s correct pronunciation can be derived from traditional orthographic-phonological relationships based on a word’s spelling on a Likert scale from 1 to 6 (1 = very easy to match/highly transparent, 6 = very difficult to match/highly opaque). Words above the mean rating (2.58) were coded as 1 (i.e., relatively low transparency), and those below the mean were coded as 0 (i.e., relatively high transparency). Target-stimulus PLD = phonological Levenshtein distance between the target word and the stimulus (the mispronunciation) in the set for variability task.

SDA = successful decoding attempts; Transparency = spelling-to-pronunciation transparency rating; OLD20 = average orthographic Levenshtein distance to 20 closest orthographic neighbors; HAL = hyperspace analogue to language; PO = phonological-orthographic; PLD20 = average phonological Levenshtein distance to 20 closest phonological neighbors; Target-stimulus PLD = phonological Levenshtein distance between the target word and the stimulus (the mispronunciation) in the set for variability task.

### Appendix B

**Table:**

Word	Prior SDA dependence	Transparency	*N* Letters	OLD20	*N* Phonemes	*N* Syllables	PLD20	Concreteness	Semantic Diversity	*N*Phono- graphic Neighbors	Log HAL Frequency	P-O Surprisal	Target-Stimulus PLD
ache	1	3.39	4	1.65	2	1	1	3.43	1.40	0	6.74	2.19	2
blind	0	1.85	5	1.70	5	1	1.75	4.03	1.95	3	9.51	0.34	1
body	0	1.89	4	1.70	4	2	1.25	4.79	1.92	0	11.66	1.56	2
break	1	2.26	5	1.65	4	1	1.2	3.71	2.15	0	10.77	3.94	1
camel	0	1.50	5	1.85	4	2	1.75	4.93	1.65	0	8.12	0.86	2
chemist	1	3.50	7	2.45	6	2	2.3	4.24	1.37	0	7.48	0.81	1
chorus	1	3.65	6	1.90	5	2	1.25	4.03	1.50	0	8.7	1.86	4
deaf	1	2.37	4	1.75	3	1	1.4	3.27	1.09	1	8.72	3.94	1
deny	1	2.46	4	1.75	4	2	1.85	2.2	1.95	1	9.48	1.56	2
devil	0	1.92	5	1.90	4	2	1.8	3.41	1.62	0	9.02	2.30	2
iron	1	2.00	4	1.70	3	1	1.85	4.59	1.74	0	9.86	1.12	2
island	0	3.59	6	1.90	5	2	1.8	4.96	1.56	0	10.71	8.35	4
kind	0	1.30	4	1.45	4	1	1.35	2.07	2.06	5	11.79	2.51	1
lamb	1	2.43	4	1.65	3	1	1	4.97	1.55	4	8.14	0.09	2
lizard	0	1.88	6	2.50	5	2	1.75	4.68	1.39	1	8.65	3.10	2
measles	1	2.89	7	2.40	5	2	1.95	4.69	1.17	0	7	3.94	2
metal	1	1.46	5	1.75	4	2	1.2	4.87	1.65	1	10.24	2.30	3
money	0	1.96	5	1.75	4	2	1	4.54	1.96	1	12.25	5.62	1
mystery	0	2.56	7	2.45	7	3	2.5	2.33	1.86	0	9.23	3.30	1
ninth	0	2.92	5	2.00	4	1	1.9	3.57	1.69	0	7.56	0.34	1
onion	1	3.17	5	1.85	5	2	2.2	4.86	0.81	0	7.86	2.25	3
piano	0	2.54	5	1.95	5	3	2.1	4.9	1.19	0	9.13	1.12	1
pigeon	1	3.65	6	2.75	5	2	2.4	4.71	1.75	0	6.8	2.30	3
post	0	1.32	4	1.25	4	1	1.2	4.3	2.08	5	12.53	0.39	1
prove	1	2.39	5	1.50	4	1	1.65	1.83	2.17	0	10.36	3.02	1
pudding	0	2.35	7	1.85	5	2	1.55	4.9	1.29	1	7.13	7.63	2
rely	1	2.04	4	1.90	4	2	1.85	1.93	2.11	0	9.03	1.56	2
rhythm	0	3.96	6	3.30	5	2	2.05	3.07	1.46	0	8.49	13.84	1
river	0	1.72	5	1.55	4	2	1.55	4.89	1.54	5	10.21	1.12	1
scent	1	3.36	5	1.70	4	1	1	3.97	1.50	0	7.8	0.55	1
scissors	0	3.48	8	3.30	5	2	1.8	4.85	1.54	0	7.22	8.97	1
soup	1	2.77	4	1.70	3	1	1	4.72	1.49	2	8.71	0.58	1
spinach	0	3.04	7	2.90	6	2	2.75	4.77	0.99	0	6.8	0.81	1
stomach	1	3.52	7	2.90	6	2	2.15	4.89	1.62	0	8.95	0.81	3
tongue	1	3.30	6	2.20	3	1	1.15	4.93	1.57	0	9.61	5.32	4
treasure	0	2.48	8	2.25	5	2	1.95	4.32	1.66	0	8.41	5.81	2
veins	1	2.63	5	1.85	4	1	1.25	N/A	1.45	2	7.27	7.62	1
wasp	1	1.88	4	1.65	4	1	1.95	4.96	1.38	2	6.67	0.39	1
weather	0	2.76	7	1.80	4	2	1.5	3.83	1.74	4	9.97	3.94	1
whom	1	2.85	4	1.80	3	1	1.05	1.93	2.11	0	10.25	9.30	2

Note. 1 = dependent on prior SDA, 0 = not dependent on prior SDA.
