# Supplementary material for: Is Set for Variability Performance Dependent on Prior Successful Decoding Attempts for Certain Items? An Item-Level Examination
Source: Sci Stud Read. Author manuscript; Available in PMC 2026 Nov 3. (PMC13727300; doi:10.1080/10888438.2026.2704734)
Supplement: Supplementary file 1 [file NIHMS2207169-supplement-Supp_1.docx]

Supplemental Materials

Table S1. *Main effects of word features on probability of correct responses in the set for variability (SfV) task.*

|  | Model I | | | | Model II | | | |
| --- | --- | --- | --- | --- | --- | --- | --- | --- |
| Fixed effects | Est. | *SE* | *z* | *p* | Est. | *SE* | *z* | *p* |
| **Intercept** | **-1.00** | **.20** | **-4.93** | **<.01** | -.28 | .29 | -.97 | .33 |
| **Word-Level Factors^a^** |  |  |  |  |  |  |  |  |
| Prior SDA dependence | – | – | – | – | **-1.37** | **.42** | **-3.22** | **<.01** |
| P-O surprisal | .12 | .07 | 1.78 | .08 | .04 | .07 | .60 | .55 |
| PLD20 | **1.10** | **.45** | **2.44** | **.01** | .59 | .43 | 1.36 | .18 |
| Target-stimulus PLD | -.23 | .23 | -1.02 | .31 | -.11 | .21 | -.51 | .61 |
| Transparency | -.39 | .32 | -1.20 | .23 | .03 | .32 | .09 | .93 |
| Intercepts  Word | Variance | | Variance Explained | | Variance | | Variance Explained | |
|  | 1.53 | | 20.43% | | 1.21 | | 37.01% | |
| *Note*. Est.= Parameter Estimate; *SE* = Standard Error; SDA = Successful Decoding Attempts; P-O = Phonological-orthographic; PLD20 = average Phonological Levenshtein Distance to 20 closest phonological neighbors; Target-stimulus PLD = Phonological Levenshtein Distance between the target word and the stimulus (the mispronunciation) in the set for variability task; Transparency = Spelling-to-Pronunciation Transparency Rating.  Each of the predictors and respective estimates represent the results from predicting probability of SfV accuracy from all variables simultaneously (i.e., in the presence of all other word-level predictors in the model). ^a^Word-level factors represent fixed characteristics of the 40 words that remain constant across individual participants in the SfV task. | | | | | | | | |

| Table S2. *Main effects of alternate coding versus dependence on prior successful decoding attempt (SDA) on probability of correct responses in the set for variability (SfV) task* | | | | | | | | | | | | |  |
| --- | --- | --- | --- | --- | --- | --- | --- | --- | --- | --- | --- | --- | --- |
|  | Mean Split Transparency Model^a^ | | | | Prior SDA Dependence Model | | | | Target-Stimulus PLD Model | | | |  |
| Fixed effects | Est. | *SE* | *z* | *p* | Est. | *SE* | *z* | *p* | Est. | *SE* | *z* | *p* |  |
| **Intercept** | **-.70** | **.27** | **-2.56** | **.01** | -.31 | .28 | -1.12 | .26 | -.59 | .26 | -2.25 | .02 |  |
| **Person-Level Factors^b^** |  |  |  |  |  |  |  |  |  |  |  |  |  |
| PDE | **.02** | **<.01** | **5.96** | **<.01** | **.02** | **<.01** | **4.18** | **<.01** | **.02** | **<.01** | **4.18** | **<.01** |  |
| PA | **.04** | **.01** | **6.72** | **<.01** | **.04** | **.01** | **6.54** | **<.01** | **.04** | **.01** | **6.54** | **<.01** |  |
| Vocabulary | **.03** | **.01** | **5.18** | **<.01** | **.03** | **.01** | **4.79** | **<.01** | **.02** | **.01** | **4.79** | **<.01** |  |
| Word Reading | **.05** | **<.01** | **9.74** | **<.01** | **.06** | **.01** | **10.38** | **<.01** | **.06** | **.01** | **6.54** | **<.01** |  |
| **Word-Level Factors^c^** |  |  |  |  |  |  |  |  |  |  |  |  |  |
| Frequency | .08 | .15 | .50 | .61 | .05 | .14 | .33 | .74 | .12 | .15 | .82 | .41 |  |
| Alternate feature | -.69 | .43 | 1.60 | .11 | – | – | – | – | -.83 | .37 | -2.21 | .03 |  |
| N of Phonemes | **.61** | **.30** | **2.02** | **.04** | .30 | .29 | 1.02 | .31 | .48 | .30 | 1.59 | .11 |  |
| PLD20 | .10 | .70 | .14 | .89 | .16 | .64 | .25 | .80 | .35 | .67 | .52 | .60 |  |
| Prior SDA Dependence | – | – | – | – | **-1.32** | **.41** | **-3.19** | **<.01** | – | – | – | – |  |
| Transparency | – | – | – | – | .05 | .30 | .18 | .86 | -.17 | .30 | -.57 | .57 |  |
| Intercepts | Variance | | Variance Explained | | Variance | | Variance Explained | | Variance | | Variance Explained | |  |
| Child | 0.22 | | 79.44% | | 0.21 | | 80.40% | | 0.22 | | 80.40% | |  |
| Word | 1.42 | | 26.24% | | 1.19 | | 38.14% | | 1.33 | | 30.65% | |  |
| *Note*. Est.= Parameter Estimate; *SE* = Standard Error; SDA = Successful Decoding Attempts; PLD = Phonological Levenstein Distance; PDE = Phonemic Decoding Efficiency; PA = Phonological Awareness; HAL = Hyperspace Analogue to Language; PLD20 = average Phonological Levenshtein Distance to 20 closest phonological neighbors; Transparency = Spelling-to-Pronunciation Transparency Rating. Each of the predictors and respective estimates represent the results from predicting probability of SfV accuracy from all variables simultaneously (i.e., in the presence of all other word- and person-level predictors in the model). ^a^The key difference between the effects presented here and those in Table 7 is that no item-specific or interaction predictors are included in the models presented here, only the main effects of child- and word-level predictors. ^b^Child-level factors represent aggregate performance by the child on the measure. ^c^Word-level factors represent fixed characteristics of the 40 words that remain constant across individual participants in the set for variability task. | | | | | | | | | | | | |  |

| Table S3. *Effects of coding based on alternate word features on probability of correct responses in the set for variability (SfV) task* | | | | | | | | | | | | |
| --- | --- | --- | --- | --- | --- | --- | --- | --- | --- | --- | --- | --- |
|  | Mean Split Phonemes Model | | | | Mean Split Length Model | | | | Mean Split Frequency Model | | | |
| Fixed effects | Est. | *SE* | *z* | *p* | Est. | *SE* | *z* | *p* | Est. | *SE* | *z* | *p* |
| **Intercept** | **-2.19** | **.28** | **-7.90** | **<.01** | **-1.99** | **.27** | **-7.28** | **<.01** | **-1.74** | **.27** | **-6.56** | **<.01** |
| **Item-by-word interactions^a^** |  |  |  |  |  |  |  |  |  |  |  |  |
| Word Reading x Mean  Split Variable | -.02 | .10 | -.16 | .87 | .08 | .10 | .83 | .41 | -.04 | .10 | -.41 | .68 |
| **Item-specific Factors^b^** |  |  |  |  |  |  |  |  |  |  |  |  |
| Word Reading | **1.04** | **.07** | **14.38** | **<.01** | **1.00** | **.07** | **14.11** | **<.01** | **1.05** | **.06** | **16.21** | **<.01** |
| **Person-Level Factors^c^** |  |  |  |  |  |  |  |  |  |  |  |  |
| PDE | **.02** | **<.01** | **4.06** | **<.01** | **.01** | **<.01** | **4.06** | **<.01** | **.02** | **<.01** | **4.05** | **<.01** |
| PA | **.04** | **.01** | **6.36** | **<.01** | **.04** | **.01** | **6.34** | **<.01** | **.04** | **.01** | **6.35** | **<.01** |
| Vocabulary | **.02** | **.01** | **4.69** | **<.01** | **.02** | **.01** | **4.68** | **<.01** | **.02** | **.01** | **4.68** | **<.01** |
| Word Reading | **.04** | **.01** | **5.86** | **<.01** | **.04** | **.01** | **5.84** | **<.01** | **.04** | **.01** | **5.86** | **<.01** |
| **Word-Level Factors^d^** |  |  |  |  |  |  |  |  |  |  |  |  |
| Mean Split Variable | **1.22** | **.53** | **2.32** | **.02** | .80 | .55 | 1.45 | .15 | .09 | .41 | .23 | .82 |
| N of Phonemes | – | – | – | – | .28 | .32 | .87 | .38 | **.58** | **.28** | **2.10** | **.04** |
| PLD20 | .46 | .52 | .87 | .38 | .39 | .63 | .62 | .53 | .13 | .59 | .22 | .83 |
| Transparency | -.30 | .31 | -.97 | .33 | -.37 | .32 | -1.14 | .26 | -.19 | .29 | -.66 | .51 |
| Intercepts  Child  Word | Variance  0.22  1.17 | | | | Variance  0.22  1.15 | | | | Variance  0.22  1.24 | | | |
| *Note*. Est.= Parameter Estimate; *SE* = Standard Error; PDE = Phonemic Decoding Efficiency; PA = Phonological Awareness; HAL = Hyperspace Analogue to Language; PLD20 = average Phonological Levenshtein Distance to 20 closest phonological neighbors; Transparency = Spelling-to-Pronunciation Transparency Rating. Each of the predictors and respective estimates represent the results from predicting probability of SfV accuracy from all variables simultaneously (i.e., in the presence of all other word- and person-level predictors in the model). ^a^An item-by-word interaction represents an interaction of an item-specific response’s accuracy (varying by participant and item in the task) by a feature of a word that remains constant across all participants. ^b^Item-specific factors represent item-specific performance; ^c^Child-level factors represent aggregate performance by the child on the measure. ^d^Word-level factors represent fixed characteristics of the 40 words that remain constant across individual participants in the set for variability task. | | | | | | | | | | | | |
